# Cost-minimization analysis of sequential genetic testing versus targeted next-generation sequencing gene panels in patients with pheochromocytoma and paraganglioma

**DOI:** 10.1080/07853890.2021.1956687

**Published:** 2021-07-26

**Authors:** Weenita Pipitprapat, Oraluck Pattanaprateep, Nareenart Iemwimangsa, Insee Sensorn, Bhakbhoom Panthan, Poramate Jiaranai, Wasun Chantratita, Kinnaree Sorapipatcharoen, Preamrudee Poomthavorn, Pat Mahachoklertwattana, Thanyachai Sura, Atchara Tunteeratum, Kanoknan Srichan, Chutintorn Sriphrapradang

**Affiliations:** a Department of Medicine, Faculty of Medicine Ramathibodi Hospital, Mahidol University, Bangkok, Thailand; b Department of Clinical Epidemiology and Biostatistics, Faculty of Medicine Ramathibodi Hospital, Mahidol University, Bangkok, Thailand; c Center for Medical Genomics, Faculty of Medicine Ramathibodi Hospital, Mahidol University, Bangkok, Thailand; d Department of Pediatrics, Faculty of Medicine Ramathibodi Hospital, Mahidol University, Bangkok, Thailand

**Keywords:** Cost analysis, economic decision analysis, health economics, neuroendocrine tumours, next-generation sequencing, sequence analysis

## Abstract

**Introduction:**

Pheochromocytomas and paragangliomas (PPGLs) are highly heritable tumours, with up to 40% of cases carrying germline variants. Current guidelines recommend genetic testing for all patients with PPGLs. Next-generation sequencing (NGS) enables accurate, fast, and inexpensive genetic testing. This study aimed to compare the costs related to PPGL genetic testing between the sequential testing using the decisional algorithm proposed in the 2014 Endocrine Society guidelines and targeted NGS gene panels.

**Methods:**

Patients with proven PPGLs were enrolled. A gene list covering 17 susceptibility genes related to hereditary PPGLs was developed for targeted sequencing. Validation was carried out by Sanger sequencing. We simulated the diagnostic workflow to examine the anticipated costs based on each strategy for genetic testing.

**Results:**

Twenty-nine patients were included, among whom a germline variant was identified in 34.5%. A total of 22.7% with apparently sporadic PPGL carried a variant. Five genes were involved (*RET*, *n* = 3; *SDHB*, *n* = 3; *SDHD*, *n* = 2; *EGLN1*, *n* = 1; and *NF1*, *n* = 1). According to the diagnostic workflow, the average cost of the targeted NGS (534.7 US dollars per patient) is lower than that of the sequential testing (734.5 US dollars per patient). The targeted NGS can also reduce the number of hospital visits from 4.1 to 1 per person. The cost can be further reduced to 496.24 US dollars per person (32% reduction) if we apply a new syndromic-driven diagnostic algorithm to establish priorities for specific genetic testing for syndromic and selected cases, and targeted NGS for non-syndromic patients.

**Conclusions:**

Targeted NGS can reduce both the cost of PPGL genetic testing and the number of hospital visits, compared with the conventional approach. Our proposed algorithm is the preferred approach due to its significant reduction of the cost of genetic testing.

**Key message**
- Pheochromocytomas and paragangliomas are highly heritable neoplasms.
- The targeted next-generation sequencing (NGS) gene panels have proven to be fast, accurate, and inexpensive for the genetic analysis.
- According to this cost analysis, it is economically reasonable to use targeted NGS gene panels for genetic screening.

## Introduction

A pheochromocytoma (PCC) is a neuroendocrine tumour arising from the adrenal medullary chromaffin cells, which mostly synthesises, metabolises, stores, and secretes catecholamines. A paraganglioma (PGL) is a tumour originating along the extra-adrenal ganglia, which is further classified as parasympathetic or sympathetic. Catecholamine-secreting sympathetic PGLs are located mostly in the abdomen, less commonly in the pelvis, and rarely in the mediastinum. In contrast to their sympathetic counterparts, parasympathetic PGLs arise from the ganglia in the head and neck and rarely produce catecholamines. PCCs and PGLs display similar histopathological features and are frequently denoted as PPGLs.

Typically, PPGLs are diagnosed when patients have the classic symptoms of paroxysmal headache, palpitation, diaphoresis, and hypertension. If not properly diagnosed, hypersecretion of catecholamines may lead to devastating consequences of these tumours, such as hypertensive crisis, flash pulmonary oedema, and even multiorgan failure and death. Asymptomatic patients with PPGLs are increasingly identified, particularly in those with incidental imaging findings or having surveillance screening based on genetic risk or past history of the tumours [1].

PPGLs have the highest heritability among all human neoplasms with up to 40% of patients carrying a pathogenic germline variant [2]. More than 20 genes are linked to hereditary PPGLs [3]. Classical syndromic associations include von Hippel–Lindau disease (VHL), multiple endocrine neoplasia type 2 (MEN2), and neurofibromatosis type 1 (NF1), due to pathogenic variants in *VHL*, *RET*, and *NF1*; and familial PGL syndromes types 1–5, associated with pathogenic variants in *SDHD*, *SDHAF2*, *SDHC*, *SDHB*, and *SDHA*, respectively. Several novel pathogenic genes have been identified, but their prevalence was reported to be low. The gene-specific clinical data provide personalised approaches to diagnostics, management, follow-up, and tumour surveillance [4–6]. Genetic test results have implications not only for optimal care of the patient, but also for family members carrying the same variant. Current guidelines recommend that testing for germline variants be performed in all PPGL patients [7,8].

Genetic testing algorithms using Sanger sequencing are still effective for patients with syndromic features. However, the highly diverse clinical presentation of PPGLs is well established, and genotypes cannot always be predicted from their phenotypes. Many stepwise diagnostic algorithms have been proposed to streamline the increasingly burdensome and costly process of genetic screening of PPGLs [7]. Owing to the growing number of PPGL-related genes identified, conventional Sanger sequencing has been superseded by next-generation sequencing (NGS), using systems in which all relevant genes can be analyzed simultaneously in a single panel [9]. The diagnosis of NF1 is mostly based on the characteristic clinical features and a review of the pedigree. The genetic testing of *NF1* is complex and time-consuming due to the large gene size (∼350 kb and 60 exons), the existence of pseudogenes, the lack of a mutation hotspot, and the extreme clinical variability. However, useful mutation-specific genotype–phenotype correlations are emerging [10,11], the identification of which can be valuable in counselling, management, and surveillance. Interestingly, the use of targeted NGS gene panels has led to the discovery of pathogenic *NF1* germline mutations in patients with PCCs without a clinical suspicion of NF1 [12,13].

The targeted NGS gene panels have proven to be fast, accurate, and inexpensive for the genetic analysis of PPGLs, as demonstrated by several studies [14–16], but implementing such a strategy requires evidence from economic evaluations. However, to the best of our knowledge, no study specifically on the economic evaluation of PPGL genetic testing has been performed. Therefore, this study aimed to evaluate the cost of PPGL genetic testing using targeted NGS gene panels compared with that by conventional Sanger sequencing using the decisional algorithm proposed in the 2014 Endocrine Society guidelines [7].

## Materials and methods

### Participants

The genetic testing was performed in PPGL patients who were regularly treated at Ramathibodi Hospital during 2019–2020. All cases had been histologically confirmed as PPGLs. This study was approved by the Human Research Ethics Committee of the Faculty of Medicine Ramathibodi Hospital, Mahidol University (MURA2020/1909) and conducted in accordance with the Declaration of Helsinki. Written informed consent was obtained from each patient prior to genetic diagnosis.

### Clinical data collection

Clinical characteristics, including age at diagnosis, sex, presenting symptoms, family history of PPGL or PPGL-related tumours, catecholamine biochemical phenotype, size and location of tumour(s), presence of multiple tumours (i.e. >1 PCC or PGL, including bilateral PCCs), presence of tumour metastases in non-chromaffin organs, clinical course, and treatment, were collected from the medical records.

### DNA sequencing and data analysis

Genetic analysis was performed at our Centre for Medical Genomics. Peripheral blood samples of the patients were prospectively collected. Genomic DNA was extracted in a manner similar to that described previously [17]. To identify the variants, clinical exome sequencing was performed using the Illumina MiSeq^®^ system (Illumina, San Diego, CA) with the TruSight One Sequencing Panel^®^. The TruSight One Sequencing Panel^®^ covers 12 Mb of genomic content, including 4811 known disease-causing genes that have been reported to be associated with human diseases. Reads were aligned to the human hg19 reference genome.

We developed a custom-designed multigene panel, which covers 17 susceptibility genes related to syndromic PPGL or hereditary PPGL (*BAP1*, *EGLN1*, *EPAS1*, *FH*, *KIF1B*, *KMT2D*, *MAX*, *MEN1*, *NF1*, *RET*, *SDHA*, *SDHAF2*, *SDHB*, *SDHC*, *SDHD*, *TMEM127*, *VHL*) including exon–intron boundaries. Variant annotation was performed with VarSeq^®^ Software version 2.1.1 (Golden Helix Inc., Bozeman, MT). Variants were filtered based on in-house developed PPGL gene list and minor allele frequency (MAF) of less than 0.05 across the online database (e.g. gnomAD, 1000 Genomes, ExAC, dbSNP, and ClinVar) and in-house Thai database (455 persons). Using the American College of Medical Genetics and Genomics (ACMG) 2015 variant classification guidelines together with Varsome^®^ Software (Saphetor, Lausanne, Switzerland), the clinical interpretation of selected variants was determined. Computational and prediction data using *in silico* tools were done as one of the ACMG criteria. Variants that were classified as pathogenic or likely pathogenic were considered to be definite causes of PPGL in the patients. Variants that did not meet the criteria of pathogenic, likely pathogenic, benign, or likely benign, would be classified as variant of uncertain significance (VUS). Variants found using NGS were subsequently validated by Sanger sequencing. The genotype–phenotype correlation of PPGL was analyzed.

### Cost-minimization analysis

**Figure 1.**
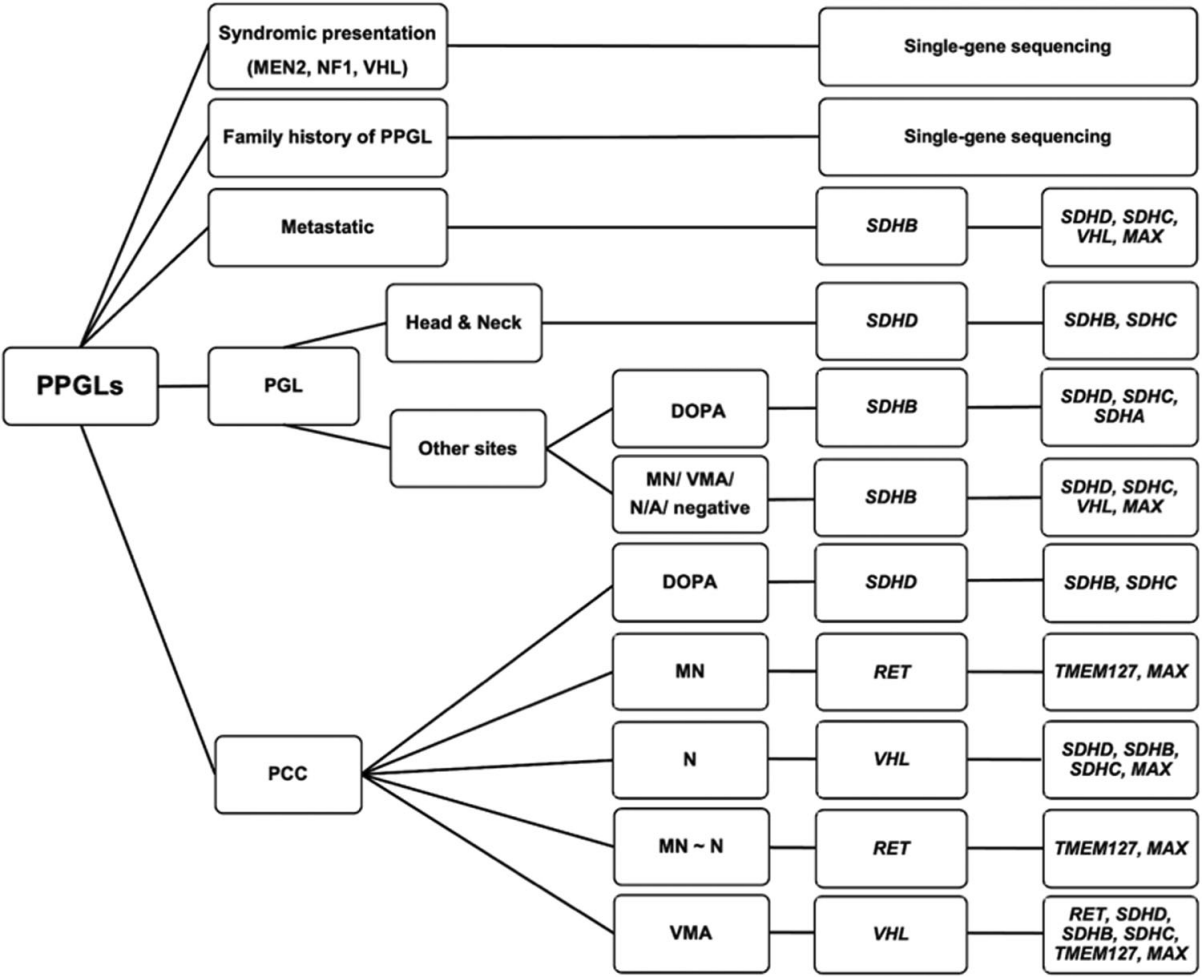
Modified decisional algorithm for genetic testing in patients with pheochromocytoma–paraganglioma based on the 2014 Endocrine Society clinical practice guidelines. Abbreviations: DOPA: dopamine; MEN2: multiple endocrine neoplasia type 2; MN: metanephrine; N: normetanephrine; NF1: neurofibromatosis type 1; N/A: not available; PCC: pheochromocytoma; PGL: paraganglioma; PPGL: pheochromocytoma–paraganglioma; VHL: von Hippel–Lindau disease; VMA: vanillylmandelic acid.

**Figure 2.**
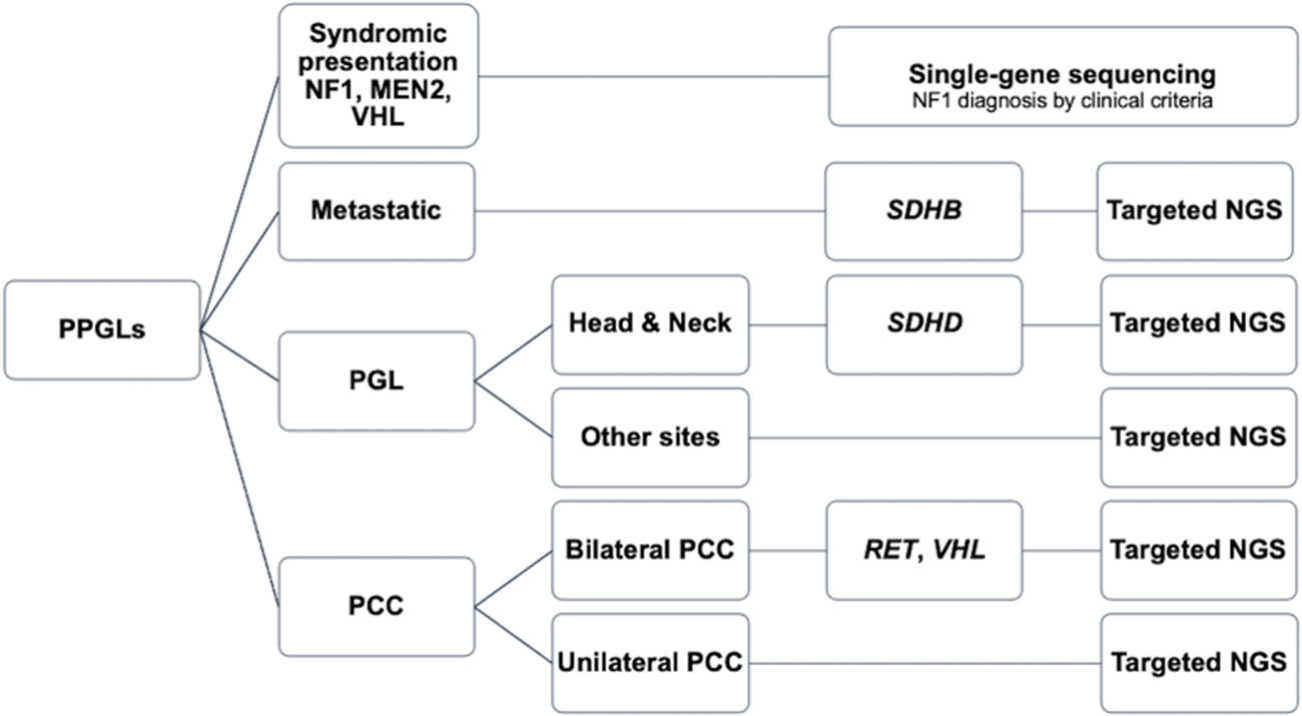
Our suggested decisional algorithm for pheochromocytoma*–*paraganglioma genetic testing. Abbreviations: MEN2: multiple endocrine neoplasia type 2; NF1: neurofibromatosis type 1; NGS: next-generation sequencing; PCC: pheochromocytoma; PGL: paraganglioma; PPGL: pheochromocytoma–paraganglioma; VHL: von Hippel–Lindau disease.

To systematically describe the costs associated with PPGL genetic testing, we simulated the diagnostic workflow on the basis of sequential single-gene testing from published clinical practice guidelines for the genetic testing of PPGLs [7], as well as targeted NGS gene panels (as described above), and our new proposed approach. As the first approach for PPGL genetic testing to be analyzed for its cost, the 2014 Endocrine Society clinical practice guidelines proposed a decisional algorithm for sequential genetic testing with the selection of genes to be tested prioritised according to a syndromic or metastatic presentation, tumour location, and catecholamine biochemical phenotype [7]. For this study, the algorithm was redesigned (Figure 1) because some decisions were not covered by the original version, such as for PPGL patients with elevated urinary vanillylmandelic acid (VMA), elevations of both noradrenaline and adrenaline, or pathologically confirmed PPGLs without evidence of elevated catecholamine. Assumptions based on the prevalence data from the literature [18] and expert opinion, were used to modify the decisional algorithm. The second approach for PPGL genetic testing to be analyzed for its cost is the targeted NGS gene panels that are widely used for patients with PPGLs. In addition, we also analyzed the cost of our PPGL genetic testing approach newly proposed here, which initially prioritises single-gene sequencing for syndromic cases (MEN2A and VHL). In this new approach, for patients with metastatic disease, *SDHB* should be tested first as hereditary *SDHB*-related PPGLs have the highest metastatic potential (up to 71% of affected patients) [2,19]. For patients with bilateral PCCs, genetic screening of *VHL* and *RET* should be the priority as approximately 19–35% and 52–61% of these patients were reported to have *VHL* or *RET* mutations, respectively [20–22]. Head and neck PGLs are more likely to be caused by the mutation of *SDHD* [19]. In cases with obvious clinical features of NF1, no genetic testing is needed. Furthermore, in this new approach, targeted NGS gene panels are performed in the non-syndromic cases (Figure 2).

Cost-minimization analysis was performed in order to compare the costs of each strategic approach from a societal perspective, consisting of direct and indirect costs. The direct cost includes medical costs (e.g. laboratory costs) and non-medical costs (e.g. costs of transportation and meals, and caregivers’ time). The indirect cost includes the lost productivity of patients. Laboratory costs were based on the costs of single-gene sequencing and targeted NGS gene panels. Costs of services were based on the costs provided by the Ramathibodi Hospital Laboratory. The direct non-medical cost and indirect cost (Table 1) were obtained with reference to the 2009 standard cost lists for economic evaluation of health in Thailand [23]. All costs were converted to 2020 values using the Thai consumer price index (Bureau of Trade and Economic Indices, 2020) [24]. Costs are expressed in 2020 baht. The exchange rate was approximately 30 baht/US$ [25].

**Table 1.** Overall direct and indirect costs (in US dollars) involved in pheochromocytoma–paraganglioma genetic testing.

Costs	US dollars
**Direct medical costs**	
Single-gene sequencing	66.67–300
Targeted NGS gene panel	509.67
Targeted NGS gene panel including Sanger sequencing confirmation	543
**Direct non-medical costs^a^:** meals, transportation, unpaid caregivers’ time	11.22^b^
**Indirect costs^a^:** unpaid patients’ time	3.1^b^

NGS: next-generation sequencing.

^a^Data from 2009 standard cost lists for economic evaluation of health in Thailand [23].

^b^Adjusted with Thai consumer price index year 2020 (Bureau of Trade and Economic Indices, 2020) [24].

Although single DNA extraction can provide sufficient high-quality DNA for all subsequent analyses, post-test counselling generally requires a hospital visit. In addition, the coverage for genetic analysis is not automatically funded by the government or insurance in our current healthcare system.

### Statistical analysis

Demographic data were analyzed by descriptive statistics analysis using IBM SPSS Statistics, Version 27.0 (IBM Corp, Armonk, NY). Continuous variables are presented as mean ± standard deviation (SD) and categorical variables are presented as percentages. The costs of genetic variant analysis by each approach were evaluated by the cost-minimization analysis as described above.

## Results

### Clinical characteristics

A total of 29 patients with histologically confirmed PPGLs were included in this study. Their clinical characteristics are presented in Table 2. The mean age at first diagnosis was 43.7 ± 15.7 years and 21 (72.4%) of the patients were female. Most patients (82.8%) presented with symptoms. Sixteen patients (55.2%) were diagnosed with hypertension.

**Table 2.** Clinical characteristics (*N* = 29).

Female, *n* (%)	21 (72.4%)
Mean age at diagnosis, years	43.7 ± 15.7
Symptomatic, *n* (%)	24 (82.8%)
Palpitation	12 (41.4%)
Sweating	6 (20.7%)
Headache	5 (17.2%)
Pain	5 (17.2%)
Paroxysm	4 (13.8%)
Palpable mass	3 (10.3%)
Hypertension, *n* (%)	16 (55.2%)
Diagnosis, *n* (%)	
Unilateral PCC	15 (51.7%)
Bilateral PCCs	1 (3.5%)
Single PGL	7 (24.1%)
Multifocal PGLs	3 (10.3%)
Combined PCC and PGL	1 (3.5%)
Metastatic PGL	2 (6.9%)
	
Mean tumour size, cm	4.7 ± 2.1
Biochemical tests (24-h urine collection)	
PCC, *n* (%)	
Metanephrines	4 (13.8%)
Normetanephrines	7 (24.1%)
Vanillylmandelic acid	2 (6.9%)
Both metanephrines and normetanephrines	2 (6.9%)
No biochemical test before surgery	1 (3.5%)
PGL, *n* (%)	
Normetanephrines	6 (20.7%)
Vanillylmandelic acid	2 (6.9%)
Negative	2 (6.9%)
No biochemical test before surgery	2 (6.9%)
Combined PCC and PGL, *n* (%)	
Normetanephrines	1 (3.5%)
Treatment, *n* (%)	
Tumour removal	25 (86.2%)
Tumour removal with MIBG treatment	2 (6.9%)
MIBG treatment	1 (3.5%)
MIBG treatment with chemotherapy	1 (3.5%)
Results of treatment, *n* (%)	
Remission	25 (86.2%)
No remission	3 (10.3%)
Death	1 (3.5%)
Gene variant, *n* (%)	10 (34.5%)
*RET*	3 (10.3%)
*SDHB*	3 (10.3%)
*SDHD*	2 (6.9%)
*EGLN1*	1 (3.5%)
*NF1*	1 (3.5%)
Family history of PPGLs, *n*	4 (13.8%)

MIBG: metaiodobenzylguanidine; PCC: pheochromocytoma; PGL: paraganglioma; PPGL: pheochromocytoma–paraganglioma.

The mean tumour size was 4.7 ± 2.1 cm. According to the location of tumours, 16 (55.2%) patients had only PCC, 12 patients (41.4%) had only PGL, and 1 patient (3.5%) had a combination of PCC and PGL. Five patients (17.3%) had multiple tumours and one of them had bilateral PCC. Only 13.8% of patients had a known family history of PPGL or associated clinical syndrome.

### Genetic variant analysis

**Figure 3.**
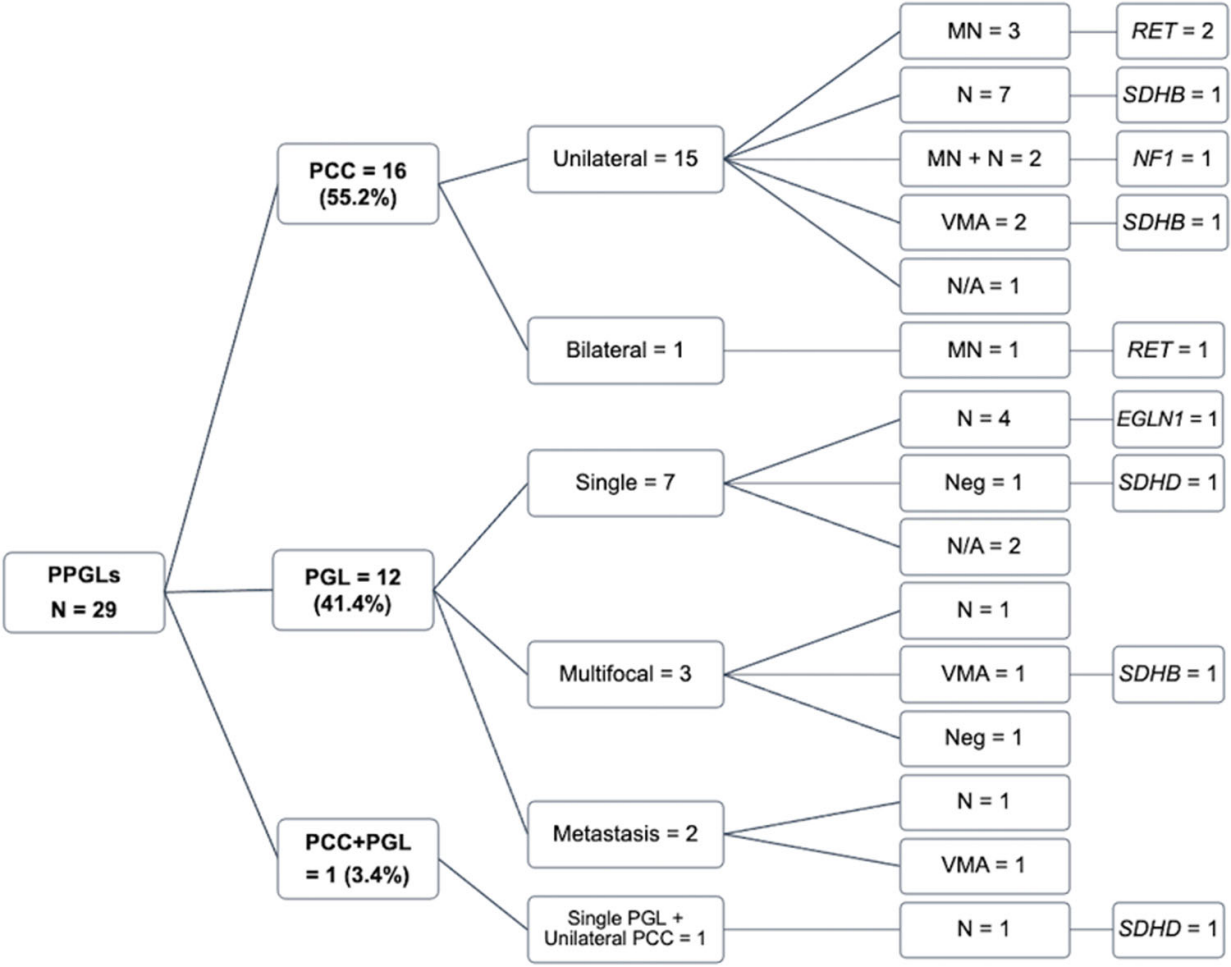
A flowchart showing baseline characteristics of pheochromocytoma–paraganglioma subtypes and genetic variants. Abbreviations: MEN2: multiple endocrine neoplasia type 2; MN: metanephrine; N: normetanephrine; NF1: neurofibromatosis type 1; N/A: not available; PCC: pheochromocytoma; PGL: paraganglioma; PPGL: pheochromocytoma–paraganglioma; VMA: vanillylmandelic acid.

Ten patients (34.5%) had a variant identified in a known susceptibility gene (Table 3). The most frequently mutated genes were *RET* (10.3%) and *SDHB* (10.3%), followed by *SDHD* (6.9%), *EGLN1* (3.4%), and *NF1* (3.4%). The details of the variants are provided in the Table 4. Figure 3 shows the distribution of germline genetic variants based on PPGL location and biochemical testing. In patients who had either a positive family history of PPGLs [MEN2A with *RET* variants, *n* = 2 (patient no. 1 and 2); familial head and neck PGL with *SDHD* variant, *n* = 2 (patient no. 7 and 8)] or an associated clinical syndrome [NF1, *n* = 1 (patient no. 10 with confirmed *NF1* variant)], the variant detection rate was 100%. Two patients (6.9%) had metastatic PGLs. None of them had an identifiable germline variant. In this study, a germline variant rate was found in 5 of the 22 patients (22.7%) among apparently sporadic PPGL patients who fulfilled the following criteria [1]: the absence of a family history [2], syndromic features [3], bilateral disease, and [4] metastatic disease.

**Table 3.** Details of the clinical characteristics.

No.	Dx	Symptoms	Urinary metabolites	No. of PPGLs	Location	Remission	Family Hx	Variants
1	Uni PCC	No	MN	1	Lt adrenal	Yes	Yes^a^	*RET* ^a^
2	Bi PCCs	No	MN	2	Rt adrenal, Lt adrenal	Yes		*RET* ^a^
3	Uni PCC	Yes	MN	1	Rt adrenal	Yes	No	*RET*
4	Uni PCC	Yes	VMA	1	Rt adrenal	Yes	No	*SDHB*
5	Uni PCC	Yes	NN	1	Lt adrenal	Yes	No	*SDHB*
6	Multi PGLs	Yes	VMA	2	Lt aortic bifurcation, Lt renal v	Yes	No	*SDHB*
7	Neck PGL, Uni PCC	Yes	N	2	Lt carotid space, Lt adrenal	Yes	Yes^b^	*SDHD* ^b^
8	Neck PGL	Yes	Neg	1	Rt carotid space	Yes		*SDHD* ^b^
9	PGL	Yes	N	1	Suprarenal	Yes	No	*EGLN1*
10	Uni PCC	Yes	MN/N	1	Lt adrenal	Yes	No	*NF1*
11	Uni PCC	Yes	MN	1	Lt adrenal	Yes	No	Not found
12	Uni PCC	Yes	N	1	Rt adrenal	Yes	No	Not found
13	Uni PCC	Yes	N	1	Lt adrenal	Yes	No	Not found
14	Uni PCC	Yes	MN/N	1	Rt adrenal	Yes	No	Not found
15	Uni PCC	Yes	N	1	Lt adrenal	Yes	No	Not found
16	Uni. PCC	Yes	N/A	1	Lt intrarenal	Yes	No	Not found
17	Uni PCC	Yes	N	1	Rt adrenal	Yes	No	Not found
18	Uni PCC	Yes	VMA	1	Rt adrenal	Yes	No	Not found
19	Uni PCC	Yes	N	1	Lt adrenal	Yes	No	Not found
20	Uni PCC	Yes	N	1	Rt adrenal	Yes	No	Not found
21	PGL	Yes	N	1	Retroperitoneum	Yes	No	Not found
22	PGL	No	N	1	Retroperitoneum	Yes	No	Not found
23	PGL	No	N	1	Retroperitoneum	No	No	Not found
24	PGL	No	N/A	1	Urinary bladder	No	No	Not found
25	PGL	Yes	N/A	1	Intradural extramedullary	Yes	No	Not found
26	Multi PGLs	Yes	N	2	Bladder, Rt para-bladder	Yes	No	Not found
27	Multi PGLs	Yes	Neg	2	Urinary bladder	Yes	No	Not found
28	Metas PGLs	Yes	VMA	1	Retroperitoneum	No	No	Not found
29	Metas PGLs	Yes	N	Multiple	Intraabdomen	Dead	No	Not found

Bi: bilateral; Dx: diagnosis; F: female; family Hx: history of first-degree relative with pheochromocytoma–paraganglioma; Lt: left; M: male; MN: metanephrine; Metas: metastatic; Multi: multifocal; N: normetanephrine; N/A: not available; Neg: negative; PCC: pheochromocytoma; PGL: paraganglioma; PPGL: pheochromocytoma–paraganglioma; Rt: right; Uni: unilateral; v: vein; VMA: vanillylmandelic acid.

^a^Familial MEN2A;

^b^Familial SDHD.

**Table 4.** Variants identified in this study (patient numbers are listed according to Table 3).

Patients no.	Gene	Zygosity	Variant (coding DNA)	Variants (protein)	Effects	Variant class
1	*RET*	Heterozygous	c.1901G > A	p.Cys634Tyr	Missense variant	Pathogenic
2	*RET*	Heterozygous	c.1901G > A	p.Cys634Tyr	Missense variant	Pathogenic
3	*RET*	Heterozygous	c.1253G > A	p.Arg418Gln	Missense variant	VUS
4	*SDHB*	Heterozygous	c.440A > G	p.Tyr147Cys	Missense variant	VUS
5	*SDHB*	Heterozygous	c.268C > T	p.Arg90Ter	Nonsense variant	Pathogenic
6	*SDHB*	Heterozygous	c.718_719delCT	p.Leu240Ilefs*15	Frameshift deletion variant	Pathogenic
7	*SDHD*	Heterozygous	c.3G > C	p.Met1Ile	Initiator codon variant	Pathogenic
8	*SDHD*	Heterozygous	c.3G > C	p.Met1Ile	Initiator codon variant	Pathogenic
9	*EGLN1*	Heterozygous	c.245C > T	p.Ala82Val	Missense variant	VUS
10	*NF1*	Heterozygous	c.3113 + 2dupT	?	Splice region variant	Pathogenic

VUS: variant of uncertain significance.

### Cost-minimization analysis

On the basis of this patient cohort, we performed a cost-minimization analysis (Tables 5 and 6) comparing single-gene sequencing according to the modified decisional algorithm proposed in the 2014 Endocrine Society guidelines (Figure 1) with targeted NGS gene panels. One patient with NF1 were excluded because the syndrome was diagnosed on the basis of the clinical criteria. From the simulation model (Tables 5 and 6), it would reduce both the number of hospital visits and the costs if the PPGL patient cohort were screened with initially targeted NGS gene panels rather than using a single-gene sequencing decision algorithm. The numbers of hospital visits associated with the use of single-gene sequencing and targeted NGS gene panels were determined to be 4.1 and 1 visit per patient, respectively. The overall costs (in Thai baht; US$1 = 30 Thai baht) of single-gene sequencing and a targeted NGS gene panel are 22,034.10 ($734.47) and 16,040.86 ($534.69) baht per patient, respectively. For the genetic screening of 28 PPGL patients, the use of targeted NGS gene panels results in a total cost reduction of 167,811.3 baht ($5593.71) or 5993.1 baht ($199.77) per patient, compared with the use of single-gene sequencing (Table 8).

**Table 5.** Sequential genetic analysis of pheochromocytoma–paraganglioma by single-gene sequencing compared with targeted next-generation sequencing (NGS) gene panels.

No.	Diagnosis	Urinary metabolites	Decisional algorithm by single-gene sequencing								Targeted NGS gene panels	
			No. of hospital visits									
			1	2	3	4	5	6	7	Total visits	Variants	Total visits
1	Uni PCC	MN	*RET*							1	*RET*	1
2	Bi PCCs	MN	*RET*							1	*RET*	1
3	Uni PCC	MN	*RET*							1	*RET*	1
4	Uni PCC	VMA	*VHL*	*RET*	*SDHD*	*SDHB*				4	*SDHB*	1
5	Uni PCC	N	*VHL*	*SDHD*	*SDHB*					3	*SDHB*	1
6	Multi PGLs	VMA	*SDHB*							1	*SDHB*	1
7	Neck PGL, Uni PCC	N	*SDHD*							1	*SDHD*	1
8	Neck PGL	Neg	*SDHD*							1	*SDHD*	1
9	PGL	N	*SDHB*	*SDHD*	*VHL*	*SDHC*	*MAX*	*EGLN1*		6	*EGLN1*	1
10	Uni PCC	MN/N	*NF1* was diagnosed based on clinical criteria									
11	Uni PCC	MN	*RET*	*TMEM127*	*MAX*					3	Not found	1
12	Uni PCC	N	*VHL*	*SDHD*	*SDHB*	*SDHC*	*MAX*			5	Not found	1
13	Uni PCC	N	*VHL*	*SDHD*	*SDHB*	*SDHC*	*MAX*			5	Not found	1
14	Uni PCC	MN/N	*RET*	*TMEM127*	*MAX*					3	Not found	1
15	Uni PCC	N	*VHL*	*SDHD*	*SDHB*	*SDHC*	*MAX*			5	Not found	1
16	Uni PCC	N/A	*VHL*	*RET*	*SDHD*	*SDHB*	*SDHC*	*TMEM127*	*MAX*	7	Not found	1
17	Uni PCC	N	*VHL*	*SDHD*	*SDHB*	*SDHC*	*MAX*			5	Not found	1
18	Uni PCC	VMA	*VHL*	*RET*	*SDHD*	*SDHB*	*SDHC*	*TMEM127*	*MAX*	7	Not found	1
19	Uni PCC	N	*VHL*	*SDHD*	*SDHB*	*SDHC*	*MAX*			5	Not found	1
20	Uni PCC	N	*VHL*	*SDHD*	*SDHB*	*SDHC*	*MAX*			5	Not found	1
21	PGL	N	*SDHB*	*SDHD*	*VHL*	*SDHC*	*MAX*			5	Not found	1
22	PGL	N	*SDHB*	*SDHD*	*VHL*	*SDHC*	*MAX*			5	Not found	1
23	PGL	N	*SDHB*	*SDHD*	*VHL*	*SDHC*	*MAX*			5	Not found	1
24	PGL	N/A	*SDHB*	*SDHD*	*VHL*	*SDHC*	*MAX*			5	Not found	1
25	PGL	N/A	*SDHB*	*SDHD*	*VHL*	*SDHC*	*MAX*			5	Not found	1
26	Multi PGLs	N	*SDHB*	*SDHD*	*VHL*	*SDHC*	*MAX*			5	Not found	1
27	Multi PGLs	Neg	*SDHB*	*SDHD*	*VHL*	*SDHC*	*MAX*			5	Not found	1
28	Metas PGLs	VMA	*SDHB*	*SDHD*	*VHL*	*SDHC*	*MAX*			5	Not found	1
29	Metas PGLs	N	*SDHB*	*SDHD*	*VHL*	*SDHC*	*MAX*			5	Not found	1
**Total no. of visits**										114		28
**No. of visits per person**										4.1		1

Bi: bilateral; MN: metanephrine; Metas: metastatic; Multi: multifocal; N: normetanephrine; N/A: not available; Neg: negative; PCC: pheochromocytoma; PGL: paraganglioma; PPGL: pheochromocytoma**–**paraganglioma; Uni: unilateral; VMA: vanillylmandelic acid.

**Table 6.** Comparison of costs of genetic analysis of pheochromocytoma–paraganglioma by sequential single-gene sequencing and targeted next-generation sequencing (NGS) gene panels.

No.	Genetic variant	Sequential single-gene sequencing					Targeted NGS gene panels				
		Total no. of visits	Direct medical cost (US$)	Direct non-medical cost (US$)	Indirect cost (US$)	Total cost (US$)	Total no. of visits	Direct medical cost (US$)	Direct non-medical cost (US$)	Indirect cost (US$)	Total cost (US$)
1	*RET*	1	66.67	11.22	3.1	80.98	1	543	11.22	3.1	557.31
2	*RET*	1	66.67	11.22	3.1	80.98	1	543	11.22	3.1	557.31
3	*RET*	1	66.67	11.22	3.1	80.98	1	543	11.22	3.1	557.31
4	*SDHB*	4	666.67	44.86	12.4	723.92	1	543	11.22	3.1	557.31
5	*SDHB*	3	600	33.65	9.3	642.94	1	543	11.22	3.1	557.31
6	*SDHB*	1	300	11.22	3.1	314.31	1	543	11.22	3.1	557.31
7	*SDHD*	1	166.67	11.22	3.1	180.98	1	543	11.22	3.1	557.31
8	*SDHD*	1	166.67	11.22	3.1	180.98	1	543	11.22	3.1	557.31
9	*EGLN1*	6	966.67	67.29	18.6	1,052.55	1	543	11.22	3.1	557.31
10	*NF1*	0	NF1 was diagnosed based on clinical criteria				0	NF1 was diagnosed based on clinical criteria			
11	Not found	3	333.33	33.65	9.3	376.28	1	509.67	11.22	3.1	523.98
12	Not found	5	866.67	56.08	15.5	938.24	1	509.67	11.22	3.1	523.98
13	Not found	5	866.67	56.08	15.5	938.24	1	509.67	11.22	3.1	523.98
14	Not found	3	333.33	33.65	9.3	376.28	1	509.67	11.22	3.1	523.98
15	Not found	5	866.67	56.08	15.5	938.24	1	509.67	11.22	3.1	523.98
16	Not found	7	1,100	78.51	21.7	1,200.2	1	509.67	11.22	3.1	523.98
17	Not found	5	866.67	56.08	15.5	938.24	1	509.67	11.22	3.1	523.98
18	Not found	7	1,100	78.51	21.7	1,200.2	1	509.67	11.22	3.1	523.98
19	Not found	5	866.67	56.08	15.5	938.24	1	509.67	11.22	3.1	523.98
20	Not found	5	866.67	56.08	15.5	938.24	1	509.67	11.22	3.1	523.98
21	Not found	5	866.67	56.08	15.5	938.24	1	509.67	11.22	3.1	523.98
22	Not found	5	866.67	56.08	15.5	938.24	1	509.67	11.22	3.1	523.98
23	Not found	5	866.67	56.08	15.5	938.24	1	509.67	11.22	3.1	523.98
24	Not found	5	866.67	56.08	15.5	938.24	1	509.67	11.22	3.1	523.98
25	Not found	5	866.67	56.08	15.5	938.24	1	509.67	11.22	3.1	523.98
26	Not found	5	866.67	56.08	15.5	938.24	1	509.67	11.22	3.1	523.98
27	Not found	5	866.67	56.08	15.5	938.24	1	509.67	11.22	3.1	523.98
28	Not found	5	866.67	56.08	15.5	938.24	1	509.67	11.22	3.1	523.98
29	Not found	5	866.67	56.08	15.5	938.24	1	509.67	11.22	3.1	523.98
Total		114	18,933.40	1278.64	353.4	20,565.18	28	14,570.73	9420.88	2603.16	14,971.41
Per person (*n* = 28)		4.1	676.19	45.67	12.62	734.47	1	520.38	336.46	92.97	534.69

NGS: next-generation sequencing.

We proposed a new approach for genetic screening in PPGL patients (Figure 2). After following our approach, the number of hospital visits was comparable to that with the initially targeted NGS gene panel (1.07 vs. 1 visit per patient), while there was a greater cost reduction of 32.44% (7146.90 baht or $238.23 per patient) vs. 27.20% (5,993.10 baht or $199.77 per patient), respectively, when compared with single-gene sequencing (Tables 7 and 8).

**Table 7.** Cost analysis of genetic testing for pheochromocytoma–paraganglioma by our proposed decisional algorithm.

No.	Diagnosis	Urinary metabolite	Syndromic/ family Hx	Suggested testing	Genetic variant	No. of hospital visits	Cost (US$)			
							Direct medical cost	Direct non-medical cost	Indirect cost	Total cost
1	Uni PCC	MN	Yes	*RET*	*RET* ^a^	1	66.67	11.22	3.1	80.98
2	Bi PCCs	MN	Yes	*RET*	*RET* ^a^	1	66.67	11.22	3.1	80.98
3	Uni PCC	MN	No	NGS	*RET*	1	543	11.22	3.1	557.31
4	Uni PCC	VMA	No	NGS	*SDHB*	1	543	11.22	3.1	557.31
5	Uni PCC	N	No	NGS	*SDHB*	1	543	11.22	3.1	557.31
6	Multi PGL	VMA	No	NGS	*SDHB*	1	543	11.22	3.1	557.31
7	Neck PGL, Uni PCC	N	Yes	*SDHD*	*SDHD* ^b^	1	166.67	11.22	3.1	180.98
8	Neck PGL	Neg	Yes	*SDHD*	*SDHD* ^b^	1	166.67	11.22	3.1	180.98
9	PGL	N	No	NGS	*EGLN1*	1	543	11.22	3.1	557.31
10	Uni PCC	MN/N	No	N/A	*NF*	0	NF1 was diagnosed based on clinical criteria			
11	Uni PCC	MN	No	NGS	Not found	1	509.67	11.22	3.1	523.98
12	Uni PCC	N	No	NGS	Not found	1	509.67	11.22	3.1	523.98
13	Uni PCC	N	No	NGS	Not found	1	509.67	11.22	3.1	523.98
14	Uni PCC	MN/N	No	NGS	Not found	1	509.67	11.22	3.1	523.98
15	Uni PCC	N	No	NGS	Not found	1	509.67	11.22	3.1	523.98
16	Uni PCC	N/A	No	NGS	Not found	1	509.67	11.22	3.1	523.98
17	Uni PCC	N	No	NGS	Not found	1	509.67	11.22	3.1	523.98
18	Uni PCC	VMA	No	NGS	Not found	1	509.67	11.22	3.1	523.98
19	Uni PCC	N	No	NGS	Not found	1	509.67	11.22	3.1	523.98
20	Uni PCC	N	No	NGS	Not found	1	509.67	11.22	3.1	523.98
21	PGL	N	No	NGS	Not found	1	509.67	11.22	3.1	523.98
22	PGL	N	No	NGS	Not found	1	509.67	11.22	3.1	523.98
23	PGL	N	No	NGS	Not found	1	509.67	11.22	3.1	523.98
24	PGL	N/A	No	NGS	Not found	1	509.67	11.22	3.1	523.98
25	PGL	N/A	No	NGS	Not found	1	509.67	11.22	3.1	523.98
26	Multi PGLs	N	No	NGS	Not found	1	509.67	11.22	3.1	523.98
27	Multi PGLs	Neg	No	NGS	Not found	1	509.67	11.22	3.1	523.98
28	Metas PGLs	VMA	No	*SDHB* followed by NGS	Not found	2	809.67	22.43	6.2	838.3
29	Metas PGLs	N	No	followed by NGS	Not found	2	809.67	22.43	6.2	838.3
**Total**						**30**	**13,465.41**	**336.58**	**93**	**13,894.73**
**Per person (*n* = 28)^c^**						**1.07**	**480.9**	**12**	**3.32**	**496.24**

Bi: bilateral; family Hx: history of first-degree relative with pheochromocytoma**–**paraganglioma; MN: metanephrine; Metas: metastatic; Multi: multifocal; N: normetanephrine; N/A: not available; Neg: negative; NGS: targeted next-generation sequencing gene panels; PCC: pheochromocytoma; PGL: paraganglioma; PPGL: pheochromocytoma**–**paraganglioma; Uni: unilateral; VMA: vanillylmandelic acid.

^a^Familial MEN2A;

^b^Familial SDHD.

^c^Excluding an NF1 patient who was diagnosed based on clinical criteria.

**Table 8.** Summary of costs by sequential single-gene sequencing, targeted next-generation sequencing (NGS) gene panels, and genetic testing followed by our proposed decisional algorithm.

				Compared with sequential single-gene sequencing (%)	
*N* = 28^a^	No. of hospital visits	Total cost (US$)	Cost per person (US$)	Total cost minimization (US$)	Cost minimization per person (US$)
Single-gene sequencing	114 (4.1/person)	20,565.18	734.47	Ref.	Ref.
Targeted NGS gene panels	28 (1/person)	14,971.47	534.7	5593.71	199.77
				**–**27.20%	
Our proposed decisional algorithm	30 (1.07/person)	13,894.76	496.24	6670.42	238.23
				**–**32.44%	

^a^Excluding an NF1 patient who was diagnosed based on clinical criteria.

Ref.: reference.

Calculating for only direct medical costs, the cost for single-gene sequencing was $676.19 per patient, while there was a greater cost reduction of 28.88% (14,427 baht or $480.90 per patient) vs. 23.04% (15,611.14 baht or $520.38 per patient) if following our approach and initially targeted NGS gene panel, respectively.

## Discussion

Because PPGLs display a high rate of heritable mutations, guidelines strongly recommend that all patients should undergo genetic testing regardless of age at diagnosis and family history [7,8]. Targeted NGS gene panels are now the most frequently performed method of genetic testing, rather than an approach using a single gene at a time [9]. This study evaluated the cost minimization of using targeted NGS gene panels in a PPGL Thai cohort. According to the decision analysis, it is economically reasonable to use targeted NGS gene panels for genetic screening. In addition, our proposed decisional diagnostic algorithm, including Sanger sequencing in syndromic and selected cases, and targeted NGS gene panels in non-syndromic cases, is more economical.

In this study, the overall variant detection rate was 34.5%. In this cohort, the most commonly mutated genes were *RET* and *SDHB*. In previous reports, mutation rates ranged from 7.5 to 22.1% in patients with apparently sporadic PPGLs [16,26–34]. In this study, the rate of germline variants in patients with sporadic PPGLs (fulfilling four criteria: the absence of a family history, syndromic features, bilateral disease, and metastatic disease) was 22.7%. According to the American Society of Clinical Oncology (ASCO) guidelines, genetic testing should be offered to individuals with a probability of carrying an inherited cancer susceptibility exceeding 10% [35,36]. Owing to this high heritability, the assessment of germline cancer susceptibility has been established as a core element of clinical practice in all patients with PPGLs, irrespective of the presence of a clear family history.

Previously, DNA samples were generally sequenced following a phenotype-driven gene prioritization by using Sanger sequencing of the coding regions of one or a few candidate genes at a time. The introduction of NGS led to a dramatic paradigm shift in molecular diagnoses for genetic conditions. Unlike Sanger sequencing, NGS is a high-throughput process that enables the massively parallel sequencing of millions of fragments simultaneously per run. Owing to the rapid increase in the number of PPGL susceptibility genes identified, NGS is perfectly suited to the genetic screening of these patients. Indeed, a consensus statement was issued by the NGS in PPGL Study Group that targeted NGS is currently the most suitable method for the genetic diagnosis of PPGLs [9]. Targeted NGS only targets the coding regions of genes associated with a specific disease using a panel. While a whole-genome sequencing (WGS) approach can capture all possible variants in both coding and non-coding regions, whole-exome sequencing (WES) or targeted NGS gene panels are more cost-effective and convenient choices for capturing phenotype-altering variants [37]. Because the genes targeted in disease NGS panels are already known to be related to specific diseases, interpretation of the sequence data is faster and more manageable than with WGS and WES. However, some practical challenges still remain regarding the interpretation of the WGS and WES data. For example, the vast majority of the genetic variations may not be directly relevant to the patient. In addition, the large number of variants incidentally discovered by WGS and WES places a burden on the diagnostic workflow and strains the process of informed consent [38].

In developing countries, the use of the NGS is limited to specialised centres. When available, the cost of testing is another barrier to the wide implementation of NGS. To reduce the cost, we proposed a prioritization approach in diagnostic genetic screening. Patients with a characteristic phenotype or family history of MEN2, VHL, and NF1 are still good candidates for targeted variant analysis by Sanger sequencing. Patients with metastatic PPGLs, head and neck PGL, and bilateral PCCs should be tested for *SDHB*, *SDHD*, and *RET*, *VHL* variants, respectively. Furthermore, targeted NGS panels should be used in the non-syndromic cases and if the previous results of suspected gene variants were negative. However, additional studies are required to assess the value of our proposed algorithm as a standard clinical application.

As health care spending has increased and resources have become more limited, questions may remain about their value [39]. Cost-effectiveness analysis (CEA) is most commonly used for evaluating value and refers to a method for measuring and comparing costs, harms, and benefits of different courses of action [39]. The information from CEA can assist public health decision-makers in allocating resources to the most cost-effective interventions that maximise the net health benefit. Although targeted NGS in PPGL patients had been mentioned as a cost-effective way of performing genetic screening [15], the term “cost-effective” is often misused in the literature. CEA is an economic analysis that compares the relative costs and health outcomes of different interventions. In this study, cost-minimization analysis was simply used to examine the costs of care due to the lack of prospective data on health outcomes. Costs refer to the total net expenditures and can be categorized as follows: formal and informal health care sector medical costs, as well as non-health care sector medical costs. Although the guidelines recommended genetic testing in all PPGL patients, in developing countries such as Thailand, the cost of the genetic test is not yet covered by the universal health system. Thus, we intended to design a protocol for genetic testing in PPGL patients that will be able to reduce the cost of the genetic testing as much as possible. Further studies should be conducted to assess the cost-effectiveness and to provide a full health analysis for the Thailand’s national health care system.

Our study was intended to analyze the cost of genetic screening for PPGL patients. However, there are limits to the extent to which these results can be generalized. For example, this study included a small sample size from a single institution. A previous study in the same setting found VHL patients at a rate of 45.7% [40], but this cohort included no VHL patients. In addition, the gene analyses did not include newly identified PPGL susceptibility genes, such as *MDH2*, *GOT2*, *SLC25A11*, *DLST*, *MERTK*, *MET*, *H3F3A*, *DNMT3A*, and *KIF1B*β [3]. However, alterations in these genes have been identified in very limited cases of PPGL. Our analysis simply shows that a targeted NGS gene panel can potentially reduce the cost and number of hospital visits associated with screening. Another limitation of this study is that the direct medical cost used for analysis was derived from the actual hospital charges, which may be differ from the costs in other hospitals. Additionally, there are many considerations unique to healthcare organizations in developing countries, and various cost factors may differ quite markedly from those in developed countries. This suggests the need for cost-of-living adjustment when performing cost analysis. For example, the usual expenses in Thailand are 30–40% less than inside the United States. Direct non-medical care and indirect costs were estimated from data collected throughout Thailand including rural areas. Travel costs and loss of productivity may be lower in rural than in urban areas [23].

In summary, targeted NGS gene panels improved the performances of PPGL genetic testing in terms of reducing the time needed for diagnosis, the number of hospital visits, and the cost compared with conventional single-gene sequencing. The cost can be further reduced if we apply a clinical feature-driven diagnostic algorithm to establish the priorities for specific genetic testing (MEN2, NF1, VHL; head and neck PGL; metastatic PGL; bilateral PCCs), and a targeted NGS gene panel strategy for non-syndromic patients.

## Data Availability

All relevant data are within the manuscript.
